# Editorial: Ultra-processed food addiction: moving toward consensus on mechanisms, definitions, assessment, and intervention

**DOI:** 10.3389/fpsyt.2025.1751346

**Published:** 2025-12-10

**Authors:** Jen Unwin, David A. Wiss, Adrian Soto-Mota

**Affiliations:** 1 The Collaborative Health Community, Oxford, United Kingdom; 2 Nutrition in Recovery, Los Angeles, CA, United States

**Keywords:** ultra-processed food, ultra-processed food addiction, ultra-processed food addiction treatment, food addiction assessment, food addiction intervention, food addiction abstinence or harm reduction

In the Research Topic, *Ultra-Processed Food Addiction: Moving toward Consensus on Mechanisms, Definitions, Assessment, and Intervention*, we focused on

ultra-processed food addiction (UPFA), which is a proposed disorder characterized by symptoms similar to those of substance use disorders, including cravings, tolerance, loss of control, withdrawal, and continued use despite negative consequences. A growing body of research and clinical experience exists regarding the neurobiology of this condition, how it differs from related disorders, and the clinical assessment and intervention protocols that warrant further study.

The concept of addiction-like symptoms in relation to ultra-processed food has remained controversial despite the documented prevalence (1) and known harms (1). Ongoing debates have centered on the disorder’s name, suitable definitions, and assessment protocols that distinguish it from eating disorders. Other debates concern whether the condition is behavioral (e.g., a process addiction) or substance-related, and what, if any, treatment approaches are effective and safe.

Recently, a group of international academics and clinicians specializing in UPFA completed a Delphi process to reach a consensus on the above questions. This effort was recognized at a London conference in 2024. This Research Topic encompasses the outcomes of the consensus exercise and contributions from participants and invited authors on the latest science and best practices related to UPFA, including the most recent advances in mechanisms, definitions, assessments, and interventions.

Lustig described the key debates over UPFA as a substance use disorder (SUD) for those interested in the historical and current scientific and public health arguments surrounding this controversial condition. He concluded that ‘*it is our expectation that the American Psychiatric Association and the World Health Organization will soon introduce Ultra Processed Food Addiction into the DSM-6 and ICD-11, respectively, with its own diagnostic code’.*

The article by Unwin et al. described the consensus process undertaken to establish where agreement currently lies among expert academics and clinicians in the field of food addiction. Participants found common ground around the name ‘Ultra-processed Food Use Disorder’ (UPFUD), agreeing that the condition is primarily a substance use disorder rather than a behavioral one. There was agreement that UPFUD is often but not always co-occurring with obesity and/or eating disorders; therefore, it warrants its own diagnostic code. Considering the symptoms as a SUD logically leads to the focus of treatment being abstinence from ultra-processed food consumption and other specific foods that cause behavioral manifestations. It was agreed that further research on treatment outcomes is needed.

We were, therefore, pleased to receive several contributions on treatment outcomes from interventions focusing on UPFUD. Unwin et al. and Bennet et al. described online group programs based on treating the condition from an addiction perspective. These include education about the underlying neurobiology, the importance of abstinence, and psychosocial support to achieve sustainable recovery. Their results show that for a majority of participants, abstinence is achievable and leads to significant and sustained improvements in both UPFUD symptoms and mental well-being. Further studies by Gudmudsdottir and Rynn, Saner et al., and Peirce-Thompson et al. showed that a telemedicine approach based on therapeutic carbohydrate restriction, along with 12-Step, metabolic health, and neuroscience-informed approaches, can all lead to successful interventions for UPFUD. More research comparing the efficacy of different approaches using randomized trials will be the next step in developing the evidence base.

The links between UPFUD and eating disorders were explored by Saner et al., Ifland and Brewerton providing further evidence for the distinction between the two disorders and suggesting that a treatment approach based on abstinence does not worsen eating disorder symptoms in those with UPFUD.

Similarly, research from Silva et al. and Wang et al. suggested that obesity and UPFUD are separate but related conditions. This work supports the case for UPFUD having its own classification in the DSM-6 and ICD-11, as argued by Lustig.

Tarman reminded us of the underlying mechanisms that explain why abstinence is an often-overlooked yet critical focus of treatment in UPFUD.

The findings by Wiss et al. underscored the cross-vulnerability between different substance-related addictive behaviors and the potential importance of integrating nutritional interventions into SUD treatment for those with adverse childhood experiences.

Cuaranta proposed an approach with Time-Restricted Eating (TRE) and the elimination of UPF within the framework of chrononutrition to simultaneously target the metabolic, circadian, and behavioral roots of mental health disorders, including UPFA.

Finally, Bennett et al. compiled guidance for healthcare practitioners on how to integrate the current knowledge of UPFUD into their practice. Given that the prevalence of this condition is now 14% of the adult population (2) and ultra-processed food consumption is linked to chronic metabolic conditions, such as diabetes and obesity, mental health problems, cancer, and all-cause mortality (3), clinicians need to understand how to recognize and intervene when this condition presents.

Given the growing recognition of the harms of ultra-processed foods and the increasing acknowledgement of their addictive potential, there is reason to be optimistic that a new diagnostic category of ultra-processed food use disorder (UPFUD) will eventually be established. Official recognition will lead to more research funding, wider availability of evidence-based treatments, directed public policies, and growing public awareness of the harms associated with ultra-processed foods.

## References

[1] Praxedes DRS Silva-Júnior AE Macena ML Oliveira AD Cardoso KS Nunes LO . Prevalence of food addiction determined by the Yale Food Addiction Scale and associated factors: A systematic review with meta-analysis. Eur Eat Disord Rev. (2022) 30:85–95. doi: 10.1002/erv.2878, PMID: 34953001

[2] Horsager C Meldgaard Bruun J Færk E Hagstrøm S Briciet Lauritsen M Dinesen Østergaard S . Food addiction is strongly associated with type 2 diabetes. Clin Nutr. (2023) 42:717–21. doi: 10.1016/j.clnu.2023.03.014, PMID: 36996685

[3] Lane MM Gamage E Du S Ashtree DN McGuinness AJ Gauci S . Ultra-processed food exposure and adverse health outcomes: umbrella review of epidemiological meta-analyses. BMJ. (2024) 384:e077310. doi: 10.1136/bmj-2023-077310, PMID: 38418082 PMC10899807

